# Use of Focused Assessment with Sonography in Trauma Examination Skills in the Evaluation of Non-trauma Patients

**DOI:** 10.7759/cureus.2076

**Published:** 2018-01-16

**Authors:** Parisa P Javedani, Gregory S Metzger, Jeremy R Oulton, Srikar Adhikari

**Affiliations:** 1 Emergency Medicne, Tucson Medical Center; 2 University of Arizona College of Medicine; 3 Department of Emergency Medicine, University of Arizona

**Keywords:** point-of-care ultrasound, fast, non-trauma, emergency department

## Abstract

Study objectives

Although the focused assessment with sonography in trauma (FAST) examination was initially developed for rapid evaluation of trauma patients, the basic skillset required to perform a FAST examination provides valuable information that may alter a non-trauma patient’s clinical course. The objective of this study was to determine the utility of the FAST examination in the emergency department management of non-trauma patients.

Methods

Cases in which the FAST examination was used to direct care in non-trauma patients were retrospectively reviewed. Following the completion of the patient's care, emergency physicians were asked to complete a questionnaire indicating how information from the FAST examination was utilized to direct care of their non-trauma patients.

Results

A total of 63 non-trauma cases with average age of 48 years (range 16-94 years) were enrolled. The FAST examination positively impacted care in 57/63 (90.5%) cases. In 18/63 (28.6%) cases, the patient’s ultimate disposition changed because of FAST examination findings. In 9/63 (14.3%) cases, paracentesis was avoided by obtaining a FAST examination, and in 8/63 cases (12.7%) paracentesis was performed due to FAST examination results. In 16/63 (25.4%) cases, anticipated imaging changed due to FAST examination findings and 4/63 (6.3%) cases did not receive the anticipated computed tomography (CT) scan.

Conclusions

Although initially developed for evaluation of trauma patients, the FAST examination can provide valuable information that can positively impact care in non-trauma patients. The FAST examination can provide information to determine appropriate patient disposition, obtain appropriate additional imaging, ensure timely consultation, and eliminate risk from unnecessary procedures.

## Introduction

Focused assessment with sonography in trauma (FAST) examination is routinely used in the emergency department (ED) to evaluate trauma patients. The FAST examination includes sonographic assessment of 1) pericardial area, 2) right upper quadrant, 3) left upper quadrant, and 4) pouch of Douglas [1]. The extended-FAST (e-FAST) examination which includes anterior thorax views evaluates for the presence of pneumothorax, and flank views evaluate for hemothorax, further expanding application of the FAST examination [2]. Although the FAST examination was initially developed to detect free fluid in trauma patients, the basic views of the FAST and e-FAST may provide valuable information that may alter a non-trauma patient’s clinical course. Appropriate acquisition of images and accurate interpretation of the FAST examination not only requires a thorough understanding of sonographic anatomy and anatomical variance, but also an understanding of the various pathologic processes that may be present in each quadrant of the FAST examination. Sonographers should be able to identify normal cardiac activity, epicardial fat pad, diaphragm, spine, liver, spleen, kidney, perirenal fat, urinary bladder, etc. Unfamiliarity of these structures’ normal sonographic anatomy could result in misinterpretation and inappropriate interventions. Thus, it is foundational for sonographers to learn sonographic anatomy well, in hopes to identify abnormal free fluid in between or surrounding these structures. These skills are taught and emphasized by point-of-care (POC) ultrasound educators when teaching FAST examination skills. We believe the knowledge of normal sonographic anatomy and scanning skills acquired with FAST examination training are adequate in the evaluation of some non-trauma patients. There are a number of applications where the FAST examination can be utilized in non-trauma patients, including but not limited to: 1) detection of free fluid in patients with suspected ruptured ectopic pregnancy or ruptured ovarian cyst, 2) evaluation for presence or absence of ascites in patients with suspected spontaneous bacterial peritonitis and subsequent procedural guidance if paracentesis is warranted, 3) detection of cardiac activity during cardiopulmonary resuscitation (CPR), 4) narrow the differential diagnosis of patients with shortness of breath (pleural effusion/pericardial effusion), 5) evaluation of the bladder in patients with urinary retention and guidance of subsequent foley catheter placement, 6) detection of hydronephrosis in patient with suspected nephrolithiasis, 7) urinary bladder evaluation prior to performing straight catheterization of the bladder in children, 8) evaluation for free fluid in hypotensive patients with concern for abdominal aortic aneurysm.

Despite the growing use of bedside POC ultrasound, the majority of emergency physicians (EPs) practicing in community settings have not incorporated bedside POC ultrasound into their practice. Stein et al. found only 29% of community EDs in California utilized bedside ultrasound and 24% used POC ultrasound regularly in Connecticut EDs [3,4]. Therefore, more time-consuming or expensive diagnostic methods may be used in lieu of ultrasound when evaluating non-trauma patients.

Studies have shown that physicians can demonstrate competence with the FAST examination after performing as few as 15 studies and it takes less than five minutes to perform the examination at bedside [5,6]. Learning the FAST examination can assist with rapid evaluation of non-trauma patients presenting to ED with variety of other symptoms (shortness of breath, chest pain, abdominal pain, hypotension, cardiac arrest). Use of the FAST examination in these patients can narrow differential diagnoses, change patient disposition, expedite consultation, avoid unnecessary procedures, and alter imaging needs. The objective of this study was to determine the utility of the FAST examination in the management of non-trauma patients in the ED.

## Materials and methods

Study design and setting

We retrospectively reviewed cases in which the FAST examination was used to direct care in non-trauma patients between July 2014 and February 2016. This study took place at two urban EDs: a 61-bed tertiary care ED with an annual census of approximately 80,000 and a 31-bed tertiary care ED with an annual census of approximately 30,000. Both institutions have an Accreditation Council for Graduate Medical  Education (ACGME)-accredited three-year emergency medicine (EM) residency program. One institution has an additional five-year combined emergency medicine/pediatrics (EM/PEDS) residency program and an active emergency ultrasound fellowship training program. FAST examinations performed by EM residents and attending physicians from both programs were included in the study. The study was approved by the Institutional Review Board.

Study population/inclusion criteria

EM residents and EM attending physicians utilizing any component of the FAST examination to guide clinical decision making in a non-trauma patient were asked to complete a questionnaire regarding the impact of the FAST examination on the patient’s ED management and course. EM core privileges include FAST examination, and all EM attending physicians were credentialed to perform a FAST examination prior to the start of this study. Every EM attending physician had completed a minimum of 50 FAST examinations, all of which had undergone quality assurance (QA) review prior to the start of the study.

Methods and measurement

EM residents and EM attending physicians with variable POC ultrasound expertise performed the FAST examination to guide clinical management in non-trauma patients. The FAST examinations were performed after the initial clinical assessment using either the Zonare Ultra (Zonare Medical Systems, Mountain View, California) or Philips Sparq (Philips Healthcare, Andover, Massachusetts) or Mindray M7 (Mindray Medical, Mahwah, New Jersey) ultrasound system. The low-frequency broadband curvilinear or a phased array transducer was used for the examination. The FAST examination included one or more of the following views: 1) Morrison’s pouch/right upper quadrant, 2) splenorenal/left upper quadrant, 3) pelvis, 4) pericardial, and/or 5) right or left hemithorax. All ultrasound examinations performed by residents were reviewed by the supervising EM attending physician. Furthermore, all ultrasound images obtained in the ED were reviewed for QA by faculty and fellows in the emergency ultrasound section.

Following completion of patient care in which a FAST examination was used to guide clinical decision making in a non-trauma patient, EM residents and EM attending physicians were asked to complete a questionnaire detailing how the FAST examination impacted the patient’s clinical course or directed ED management. The questionnaire verified the use of FAST examination in a non-trauma patient. Items in the questionnaire addressed the following: 1) views obtained, 2) pre-FAST and post-FAST examination differential diagnosis, imaging, consults, and anticipated disposition, 3) whether or not the FAST examination impacted ED management, 4) procedural interventions due to the FAST examination, 5) whether the FAST examination had a positive impact, negative impact, or no impact on the EM resident or EM attending physician’s management of the patient.

Outcome measures

The primary outcome was whether the FAST examination had a positive impact, negative impact, or no impact on the patient’s ED care. Secondary outcome measures included: 1) change in disposition, 2) immediate consultant involvement, 3) change in the necessity of a procedure, or 4) change in anticipated imaging due to the results of the FAST examination.

Data analysis

Descriptive statistics were used to summarize the data. Continuous data are presented as means with standard deviations and dichotomous data are presented as percent frequency of occurrence.

## Results

A total of 63 cases (35 females, 28 males) with average age of 48 years (range 16-94 years, SD = 21.16) were included in the study. The FAST examination positively impacted care in 57/63 (90.5%) cases. In 18/63 (28.6%) cases, the patient’s ultimate disposition changed because of FAST examination findings. Seven out of 63 (11.1%) cases with anticipated admission were discharged home based on FAST results and 1/63 (1.6%) with anticipated discharge required admission following the FAST examination. In 4/63 (6.3%) cases, the patient’s inpatient disposition was influenced by the FAST; in 1/63 (1.6%) case the patient went to the cardiac catheterization lab prior to admission and in 7/63 (11.1%) additional cases, patients with a ruptured ectopic pregnancy went directly to the operating room. In 9/63 (14.3%) cases, paracentesis was avoided by obtaining a FAST examination, and in 8/63 cases (12.7%) paracentesis was performed due to FAST examination results. In 16/63 (25.4%) cases, anticipated imaging changed due to results from the FAST examination and 4/63 (6.3%) cases did not receive the anticipated computed tomography (CT) scan.

## Discussion

Previous literature has demonstrated the use of FAST examination can impact clinical management, decrease use of ionizing radiation, and decrease time to operative intervention in trauma patients [1, 7-9]. To our knowledge, there is no published study evaluating the utility of the FAST examination skills in non-trauma patients. Our study results demonstrate that basic POC ultrasound skills required for performing the FAST examination can change patient disposition, provide valuable information for consultation, avoid unnecessary procedures, and change imaging needs in non-trauma patients. Learning the FAST examination can assist in narrowing the differential diagnosis in non-trauma patients presenting to the ED with undifferentiated chief complaints, help guide procedures, decrease the need for futile procedures, and aid in resuscitation.

In female patients of childbearing age with abdominal pain, hypotension or hemodynamic collapse, the FAST examination can quickly detect free fluid suggestive of a ruptured ectopic pregnancy or ovarian cyst. In a study done by Moore et al., free intraperitoneal fluid found in Morison’s pouch on bedside ultrasound in ED patients with suspected ectopic pregnancy accurately predicted the need for operative intervention, which highlights the utility of learning the FAST examination [10]. In this study, 11.1% of cases were immediately transferred to the operating room based on results of the bedside FAST examination. Performing a FAST examination can have life-saving implications in non-tertiary, non-trauma, and community EDs, as this allows the patient to be transferred to a facility with appropriate consultants, thereby minimizing the delay in definitive operative care.

A number of underlying pathologies can contribute to undifferentiated chest pain or shortness of breath—including pneumothorax, pleural effusion, pericardial effusion or cardiac tamponade—and these can be rapidly evaluated at the bedside using views from the FAST examination. Furthermore, ultrasound can provide procedural guidance for chest tube placement or pericardiocentesis. Basic FAST examination skills can be used to detect cardiac activity or reversible pathology such as pericardial effusion during CPR, which can aid in ED resuscitation. Tayal et al. found emergency echocardiography performed by EPs in patients presenting with pulseless electrical activity (PEA) detected pericardial effusion with treatable etiologies vs. PEA with ventricular standstill [11]. The most common and easily reversible causes of cardiac arrest—severe hypovolemia, tension pneumothorax, cardiac tamponade, and massive pulmonary embolus—vs. true asystole could potentially be detected on bedside ultrasound without interfering with resuscitative efforts [12-14].

Bedside FAST examination skills can also be used to evaluate patients with concern for ascites or suspected spontaneous bacterial peritonitis, as ultrasound can determine if there is a sufficiently large fluid pocket to benefit from paracentesis. In our study, paracentesis was not performed in 14.3% of patients after evaluation with the FAST examination, whereas in 12.7% the FAST examination resulted in performing a paracentesis. Our study findings correlate with a prior study done by Nazeer et al. where 25% of patients did not receive paracentesis because no ascites or insignificant amount of ascites was visualized using bedside ultrasound in the ED; 15/17 (88.2%) failed paracentesis attempts were evaluated using bedside ultrasound, and in 13/15 ascites fluid was obtained, 1/17 (5.9%) did not have enough fluid to be sampled, and 1/17 (5.9%) had no fluid visualized [15]. Additionally, bedside ultrasound provides procedural guidance to maximize patient safety during the paracentesis.

Published literature has established that bedside ultrasound can positively impact patient care. All providers in this study were asked the impact of the FAST examination on the patient’s ED management; a positive impact on patient care was noted in 90.5% cases, and no providers noted a negative impact on patient care from using the FAST examination. This highlights the need to learn the FAST examination and apply in non-trauma patients. Ultrasound training is now mandated by the Residency Review Committee of the ACGME for the training of emergency physicians. FAST examination is easy to learn, quick to perform, and can drastically improve patient management, making it an important skill for all ED physicians to master. EDs generally use guidelines set for by the American College of Emergency Physicians, which calls for 16 hours of ultrasound didactic training followed by 25 educational ultrasound examinations for each application [3]. Studies have demonstrated EPs can competently perform and interpret a FAST examination after as few as 15 studies, though others have suggested 35-75 studies may be needed to develop proficiency with performing the FAST examination [5,6,16].

Our study has a number of limitations including its retrospective nature of data and small number of patients. We attempted to reduce the bias in retrospective data collection by using a standardized data collection form. Another limitation of this study is the convenience sample design, which introduces a selection bias. No protocol was in place to perform FAST examination in non-trauma patients during the study period. FAST examinations were performed at the discretion of the treating physicians. Data were collected from questionnaires completed by the providers after the completion of the patient’s care. Medical records were not reviewed for history, physical examination findings, co-morbidities, diagnostic testing, disposition plan, hospital course, and follow-up visits; therefore, our results are dependent on the accuracy of the self-reported data, and are susceptible to error.

## Conclusions

Although initially developed for evaluation of trauma patients, the FAST examination requires basic ultrasound skills that are easily learned and takes less than five minutes to perform, but can provide valuable information for the clinical management of non-trauma patients. Results of FAST examination can aid in selecting patient disposition, obtaining appropriate additional imaging, ensuring timely consultation, and eliminating risk from unnecessary procedures.
